# Radiolabeled nanobodies as theranostic tools in targeted radionuclide therapy of cancer

**DOI:** 10.1517/17425247.2014.941803

**Published:** 2014-07-18

**Authors:** Matthias D’Huyvetter, Catarina Xavier, Vicky Caveliers, Tony Lahoutte, Serge Muyldermans, Nick Devoogdt

**Affiliations:** ^a^ Belgian Nuclear Research Center (SCK·CEN), Radiobiology Unit, Molecular and Cellular Biology Expert Group, Mol, Belgium; ^b^ Vrije Universiteit Brussel (VUB), In vivo Cellular and Molecular Imaging Laboratory (ICMI), Laarbeeklaan 103, 1090 Brussels, Belgium; ^c^ UZ Brussel, Department of Nuclear Medicine, Brussels, Belgium; ^d^ Vrije Universiteit Brussel (VUB), Cellular and Molecular Immunology, Pleinlaan 2, 1050 Brussels, Belgium+32 2 6291969; svmuylde@vub.ac.be; ^e^ Vlaams Instituut voor Biotechnologie (VIB), Structural Biology Research Center, Brussels, Belgium

**Keywords:** cancer, nanobody, nuclear imaging, radiochemistry, radionuclide, targeted radionuclide therapy, theranostics

## Abstract

***Introduction:*** The integration of diagnostic testing for the presence of a molecular target is of interest to predict successful targeted radionuclide therapy (TRNT). This so-called ‘theranostic’ approach aims to improve personalized treatment based on the molecular characteristics of cancer cells. Moreover, it offers new insights in predicting adverse effects and provides appropriate tools to monitor therapy responses. Recent findings using nanobodies emphasize their potential as theranostic tools in cancer treatment. Nanobodies are recombinant, small antigen-binding fragments that are derived from camelid heavy-chain-only antibodies.

***Areas covered:*** We review the current status of theranostic approaches in TRNT, with a focus on antibodies, peptides, scaffold proteins and emerging nanobodies. In recent years, nanobodies have been evaluated intensively for molecular imaging. In addition, novel data on TRNT using radiolabeled nanobodies for carcinomas and multiple myeloma highlight their promising opportunities in cancer treatment.

***Expert opinion:*** We trust that radiolabeled nanobodies will have a future potential as theranostic tools in cancer therapy, both for diagnosis as well as for TRNT.

## 1. Introduction

Radiation damages the DNA in the cell nucleus, thereby arresting cell proliferation. Damage to extranuclear targets and the signaling between hit and non-hit cells also play roles in cell killing. Indeed, several newly recognized responses have been classified as so-called non-targeted responses, in which biological effects are not directly related to the amount of energy deposited in the DNA of the cells that are being traversed by the radiation. A common feature is that most of these responses manifest themselves after exposure to low doses of radiation (< 0.5 Gy) or in conditions when cells have not been exposed uniformly or irradiated directly [1]. Notwithstanding, exposing a limited area of the body to an external high energy X-ray beam is the most common way to deliver radiation to cancer cells. An alternative approach of cancer irradiation is provided by targeted radionuclide therapy (TRNT). TRNT is a systemic treatment that aims to deliver cytotoxic radiation to cancer cells, with minimal exposure to healthy tissue. Two main categories of TRNT are distinguished. The first describes agents that naturally accumulate in malignant tissues like Iodine-131 (^131^I), ^131^I-MIBG and Strontium-89 (^89^Sr)-chloride accumulating in thyroid, neuroblastoma and bone, respectively (Table 1). The second category of TRNT agents interacts with tumor-associated antigens that are expressed on the cancer cell surface and are readily accessible by circulating agents. Examples are radiolabeled antibodies: Yttrium-90 (^90^Y)-ibritumomab and ^131^I-tositumomab to treat non-Hodgkin’s lymphoma and ^90^Y- and Lutetium-177 (^177^Lu)-octreotide to treat neuroblastoma (Table 1). Targeting tumor-associated antigens (unique or overexpressed) gains more interest as an alternative to, or in combination with, conventional treatments like chemotherapy, external beam radiation and surgery.

**Table 1.** **Currently approved targeted radionuclide therapies in oncology.**

**Indication**	**Product**	**Physical half-life (days)**	**Emission**	**Path length (mm)**
Thyroid cancer	^131^I	8.04	β, γ	4
Neuroblastoma	^177^Lu-octreotide	6.72	β, γ	1
Neuroblastoma	^90^Y-octreotide	2.7	β	12
Non-Hodgkin’s lymphoma	^90^Y-ibritumomab tiuxetan	2.7	β	12
Non-Hodgkin’s lymphoma	^131^I-tositumomab	8.04	β, γ	4
Liver metastases	^90^Y-microspheres	2.7	β	12
Phaeochromocytoma/neuroblastoma	^131^I-MIBG	8.04	β, γ	4
Bone metastases	^153^Sm-EDTMP	1.95	β, γ	3.1
Bone metastases	^89^Sr-chloride	50.5	β	8
Bone metastases	^223^Ra-chloride	11.4	a	0.5

The inclusion of a diagnostic radiotracer with identical or similar pharmacokinetic characteristics as the therapeutic compound, followed by a diagnostic scan to reveal the compound accumulation at the cancer lesion is a preferred and effective strategy to guide the TRNT towards a successful outcome. Diagnostic activities would thereby serve as support for dose estimation and impact the rationalization of treatments based on dose–effect relationships. Additionally, these diagnostic scans anticipate on potential adverse effects of the therapy and are helpful to monitor therapy responses in follow-up studies. The strategy whereby related compounds are employed both for diagnosis and therapy is generally referred to as ‘theranostics’ [2,3].

Here, we provide an overview of possible radionuclides used in TRNT, followed by a survey of antibodies and derived fragments or peptides and even engineered non-immunoglobulin protein scaffolds employed to provide the target specificity. Finally, the recent developments with nanobodies in TRNT are put in the spotlights.

## 2. Radionuclides in TRNT

Particles emitted during atomic decay are classified by means of linear energy transfer (LET) radiation. LET corresponds to the energy released over a certain distance. For the same absorbed dose, high LET is more cytotoxic than low LET radiation.

So far, TRNT has been predominantly explored using β-emitting radioisotopes, of which the most widely used examples are described in Table 2. The β-particles have a low LET of about 0.2 keV/μm, causing limited ionization and DNA damage involving single- or double-strand DNA breaks, base chemical modifications and protein crosslinks [4]. These types of events are repairable, so they might cause only sub-lethal damage. The relatively long range of β-particles causes energy disposition in neighboring non-targeted cells, known as the crossfire-effect. This phenomenon enables targeting heterogenic tumor tissue, because not every cell needs to be targeted. However, it might have the disadvantage of damaging adjacent healthy tissues.

**Table 2.** **Selection of radionuclides of interest for targeted radionuclide therapy.**

**Radionuclide**	**Physical half-life**	**Maximum energy (keV)**	**Range (μm)**
*β-particle emitters*			
^90^Y	64.1 h	2.284	11300
^131^I	193.0 h	606	2300
^177^Lu	161.0 h	497	1800
^67^Cu	61.9 h	575	2100
^186^Re	90.6 h	1077	4800
^188^Re	17.0 h	2120	10400
^153^Sm	46.7 h	817	5500
^89^Sr	50.5 days	1460	24000
*Auger-particle emitters*			
^125^I	60.1 days	31	20
^111^In	67.3 h	26	17
^67^Ga	78.3 h	10	3
^123^I	13.3 h	31	20
^195m^Pt	96.5 h	64	76
*α-particle emitters*			
^211^At	7.2 h	5.867	48
^212^Bi	1 h	5.870	51
^213^Bi	45.6 min	6.051	48
^225^Ac	240 h	5.830	48
^223^Ra	11.4 days	5.640	50

Adapted from [4,81].

Currently, TRNT using α-emitters receives more attention (Table 2). The α-particle consists of two protons and two neutrons, and possesses a high LET value of 50 – 230 keV/μm, that induces clusters of DNA damage like double-strand DNA breaks and base chemical modifications, which are difficult to repair. The damage is independent from dose rate, because a single hit can exert this injury. The path length of α-particles is short, so the energy deposit is limited to a few cells [4]. These properties seem to be ideal for treating small cell-burden, as is the case in micro-metastatic or minimal residual disease. ^223^Ra-chloride (Xofigo®) is the first α-emitting radioactive therapeutic agent that is approved by the FDA (in May 2013) for the treatment of castration-resistant prostate cancer and symptomatic bone metastases [5].

Auger-electron emitters produce intermediate LET values of 4 – 26 keV/μm. In general, it is accepted that these electrons need to be delivered close to the cell nucleus, as their path length is very short (Table 2). However, recent *in vitro* and *in vivo* data have shown that Auger electrons can be efficient as well when targeted to the cell membrane [4]. Notwithstanding, the identification of these radioisotopes has stimulated efforts to synthesize agents capable of internalizing into cancer cells and accumulating in their nuclei, for example, cell-penetrating peptides [6,7].

### 2.1 Radioactive iodine therapy

^131^I is used well over 50 years as a self-targeting adjuvant treatment together with surgery in thyroid cancer of both follicular and papillary types [8]. Disease relapse can be detected in early stages given the very sensitive detection of the tumor marker thyroglobulin in blood samples. To identify the localization of the lesions and confirm their iodine avidity, diagnostic scans using either ^123^I or a low activity of ^131^I can be performed. More recently, diagnostic imaging using iodine-124 (^124^I) and positron emission tomography (PET/CT) is being evaluated to increase the spatial resolution of the scan. After treatment with high activities of ^131^I (typically 1110 – 7400 MBq), whole body scintigraphy is performed to confirm the uptake in the known lesions and to demonstrate potential additional lesions that were not evident using low-activity diagnostic iodine imaging. In a subset of patients, iodine-avidity of the cancer lesions decreases over time, due to the loss of expression of the sodium iodine symporter. This loss of expression leads to resistance to ^131^I therapy, thereby considerably deteriorating the patient’s prognosis [9].

## 3. Antibody-based TRNT

### 3.1 Radionuclide-labeled antibodies

**Figure 1.**
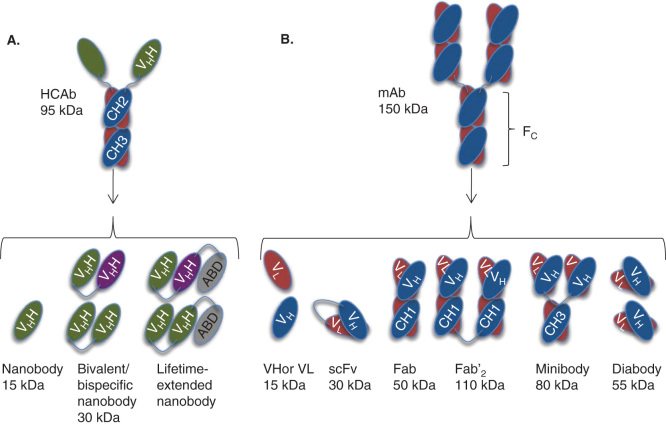
**Antibodies and their derived antigen-binding fragments.** **A.** Camelid heavy-chain-only antibody (HCAb) and its V_H_H (also known as nanobody or sdAb), bivalent and circulation-lifetime extended nanobody constructs. **B.** Conventional mAb and the derived Fab, scFv, Fv domains V_L_ or V_H_, Fab’_2_, minibody and diabody.

ABD: V_H_H or other entity binding to albumin; CH: Constant domain of an antibody; sdAb: single-domain antibody; V_H_: Variable domain of a heavy chain; V_L_: Variable domain of a light chain.

So far, the principle of TRNT has been mainly explored using mAbs as a vehicle to deliver the toxic radiation and is generally referred to as radioimmunotherapy or RIT. The mAbs are large (150 kDa) and complex molecules comprising two identical light chains and two identical heavy chains, held together by disulfide bonds. Consequently, a Y-shaped molecule is formed containing two identical antigen-binding arms (Fabs) and a glycosylated stem region (Fc) separated by a flexible hinge region (Figure 1). The Fc region is responsible for the recruitment of cytotoxic effector mechanisms, including the activation of a complement cascade and interactions with Fc-receptors on immune cells. The Fc also furnishes mAbs with an unusually long serum half-life of several days or weeks, through interactions with neonatal Fc receptors [10]. The use of mAbs is well established and currently > 150 of them are in clinical development. Today, 13 mAbs are approved by the FDA for cancer immunotherapy [11], however, mostly in unconjugated form. Their therapeutic effect is achieved by antibody-dependent cell-mediated cytotoxicity or complement-dependent cytotoxicity, and/or by interfering with the signal transduction of the targeted receptor.

The landmarks for RIT consist of the FDA approval of radiolabeled anti-CD20 mAbs ^90^Y-labeled ibritumomab tiuxetan (Zevalin®; Cell Therapeutics, Inc., Seattle, USA) and ^131^I-labeled tositumomab (Bexxar®; GlaxoSmithKline LLC, Delaware, USA), both used in the treatment of relapsed or refractory low-grade B-cell non-Hodgkin’s lymphoma. Lymphomas are classified in either Hodgkin’s (15%) or non-Hodgkin’s lymphoma (85%). Due to the high radiosensitivity of lymphomas, only a relatively low absorbed dose is required to obtain an objective response. Dose estimations are frequently done using single-photon emission computed tomography (SPECT) imaging with Indium-111 (^111^In)-labeled ibritumomab tiuxetan. Anti-CD20 RIT has proven to be successful, with 80% of the patients responding to therapy, and ∼ 30% showing a complete response [12]. Several other agents are under investigation in clinical trials for aggressive B-cell lymphomas, follicular lymphoma and acute lymphoblastic leukemia (Table 3).

**Table 3.** **Completed clinical trials of mAb-based targeted radionuclide therapies in oncology.**

**Cancer type**	**RIT agent**	**Isotopes**	**Clinical phase**	**Antigen**
Colorectal cancer				
	A5B7/A5B7 F(ab’)2	^131^I	I	CEA
	NP-4/NP-4 F(ab’)2	^131^I	I/II	CEA
	cT84.66	^90^Y	I	CEA
	F6 F(ab’)2	^131^I	I	CEA
	NR-CO-2	^186^Re	I	CEA
	COL-1*	^131^I	I	CEA
	huMN-14	^131^I	II	CEA
	CC49*	^177^Lu/^131^I/^90^Y	I	TAG-72
	cB72.3	^131^I	I	TAG-72
	A33*	^131^I/^125^I	I-I/II	A33
	huA33	^125^I	I	A33
	CO-17-1A*	^125^I	I	Ep-CAM
	NR-LU-10*	^186^Re	I	Ep-CAM
	NR-Lu-13	^186^Re	I	Ep-CAM
Breast cancer				
	cT84.66	^90^Y	I	CEA
	BrE-3*	^90^Y	I	MUC-1
	huBrE-3	^90^Y	I	MUC-1
	m170*	^90^Y	I	MUC-1
	chL6	^131^I	I	L6
	CC49*	^177^Lu	I	TAG-72
Prostate cancer				
	CC49*	^131^I	II	TAG-72
	m170*	^90^Y	I-II	MUC-1
	J591	^177^Lu/^90^Y	I	PSMA
Ovarian cancer				
	CC49 (IP)*	^177^Lu/^90^Y	I	TAG-72
	HMFG-1 (IP)*	^90^Y	I/II-III	HMFG1
	OC125 F(ab’)_2_ (IP)	^90^Y/^131^I	I-II	CA-125
	hMN-14 (IP)	^131^I	I/II	CEA
	MX35 F(ab’)_2_ (IP)	^211^At	I	NaPi2b
	TCMC-Trastuzumab (IP)	^212^Pb/^212^Bi	I	HER2
Pancreatic cancer				
	hPAM4	^90^Y	I-I/II	MUC-1
Central nervous system				
	425*	^125^I	I-II	EGFR
	ch81C6 (IRC)	^211^At/^131^I	II	TN-C
	BC4*	^90^Y	I	TN-C
Hematological malignancies				
	hLL2	^90^Y	I/II	CD22
	M195*/huM195	^131^I/^213^Bi	I-I/II	CD33
	MB-1*	^131^I	I	CD37
	BC8*	^131^I	I-I/II	CD45
	anti-CD66*	^188^Re/^90^Y	I/II-II	CD66
	Lym-1*	^131^I	I	HLA-DR
	2IT-BAT-Lym-1*	67Cu	I/II	HLA-DR
	ibritumomab tiuxetan*	^90^Y	I-I/II-II-III	CD20
	Rituximab	^131^I	I/II	CD20
	Tositumomab*	^131^I	I-II	CD20
	DOTA-Biotin/scFv 9E9-streptavidin	^90^Y	I	CD20

Agents marked with * are intact murine mAbs. Adapted from [4,82-84]. EpCAM: Epithelial cell adhesion molecule; HFMG: Human Milk Fat Globule antigen; MUC-1: Mucin 1; PSMA: Prostate-specific membrane antigen; RIT: Radioimmunotherapy; TAG-72: Tumor-associated glycoprotein 72; TN-C: Tenascin-C.

In contrast to RIT of blood-borne cancers, for larger epithelial tumor burden only limited success has been recorded so far. The majority of attempts in solid tumor-RIT have been focused on colorectal, breast, prostate, ovarian and pancreatic cancer, and some cancers of the CNS, of which the most important completed clinical studies are presented in Table 3. So far, only one Phase III trial has been completed, comparing ^90^Y-labeled murine HMFG1 (^90^Y-muHMFG1) plus standard treatment versus standard treatment alone in patients with epithelial ovarian cancer who had attained a complete clinical remission after cytoreductive surgery and platinum-based chemotherapy. The study revealed that a single intraperitoneal administration of ^90^Y-muHMFG1 to these patients did not extend survival or time to relapse, most likely due to inefficient dose estimations [13]. In general, epithelial-derived carcinomas are less radiosensitive than B-cell lymphomas and require a much higher delivered dose for clinical efficacy [14]. Unsuccessful RIT of large carcinomas can also be explained by the suboptimal pharmacokinetic properties of mAb carriers. After injection, it takes 2 – 3 days to reach maximum levels of antibody concentration within the tumor, while blood and normal tissue levels remain high. Finally, only a small fraction of the injected activity will localize in the tumor, as the large mAb size and physiological barriers prevent rapid diffusion. High-affinity mAbs are also subject to the site barrier effect, in which they are retained in the perivascular region of the tumor, in contrast to lower affinity mAbs that penetrate deeper into tumor tissue. However, the targeting efficiency of macromolecules with molecular weight above 40 kDa, such as mAbs, is further influenced by a phenomenon known as enhanced permeability and retention (EPR). Due to leaky vasculature and impaired lymph drainage, mAbs accumulate more in tumors compared to smaller molecules, and this mAb accumulation is antigen independent [15]. In an effort to reduce blood residence time, frequently mAbs used for RIT are murine and not human(-ized) (Table 3). Murine mAbs interact weaker with human Fc-receptors and are therefore cleared faster. However, unfavorable generation of human anti-murine antibodies limit repeated dosing, which indeed has been observed in case of ^90^Y-labeled ibritumomab tiuxetan. Pre-targeting is another strategy to reduce the unfavorable pharmacokinetics of mAbs. In pre-targeting, an unconjugated bispecific antibody is given in a first phase. When maximum uptake levels in the tumor are reached, in combination with a substantial clearance from non-target tissues, a small radiolabeled agent that is recognized by one arm of the bispecific antibody is given in a second phase. The latter binds to the bispecific antibody localized at the tumor, while unbound fraction is cleared via the kidneys [16]. Early dosimetry studies revealed that the dose reaching the tumor is inversely proportional to the radius of the tumor mass, making RIT mostly suitable for metastatic or minimal residual disease [17]. A Phase II clinical trial with a radiolabeled anti-CEA (carcino embryonic antigen) antibody for the treatment of colorectal cancer patients with liver metastases confirmed early preclinical studies in animal models and suggests the important potential of using TRNT as an adjuvant therapy in the management of metastatic disease [18]. In addition, encouraging results on the treatment of glioblastoma multiforme have been recorded. In this cancer type, conventional therapies like external beam radiotherapy and chemotherapy are less effective due to dose-limiting toxicity to normal brain. Radiolabeled anti-tenascin mAb 81C6, injected directly in the resection cavity after surgery led to a median survival up to 90 weeks compared to a 1-year survival for the conventional treatment methods [19].

### 3.2 Radionuclide-labeled antibody fragments

In an attempt to augment tumor penetration and fasten blood and tissue clearance, much effort has been put in the development of mAb-derived fragments [20]. Examples are V_H_ or V_L_ domains (15 kDa), scFv (30 kDa), Fab (50 kDa) and Fab’_2_ (110 kDa), as shown schematically in Figure 1. The mAb-derived fragments, having a smaller size due to the absence of an Fc-part, are cleared more rapidly through the kidneys and are increasingly being used in clinical RIT studies (Table 3). The generation of such fragments, however, frequently results in moderate affinity reagents with reduced stability, and *in vivo*, these fragments regularly show a significant degree of nonspecific accumulation in healthy tissues. As reviewed elsewhere [21], mAb-derived fragments often also suboptimally target tumor tissue, potentially due to inefficient tumor penetration. The development of diabodies (55 kDa) and minibodies (80 kDa) are efforts to combine favorable characteristics of both mAbs and mAb-derived fragments, and these constructs have shown potential in TRNT.

In conclusion, RIT beyond the treatment of B-cell non-Hodgkin lymphoma is still mostly under (pre-) clinical investigation. The prolonged blood residence time of mAbs has important implications on the level of toxicity to bone marrow (BM) and other highly perfused organs like spleen and liver, and specific accumulation of radioactivity in tumor tissue is limited. Recent advances in antibody engineering and pre-targeting strategies can at least partially compensate for these issues.

## 4. Peptide-based TRNT

An alternative strategy to target tumor-associated antigens focuses on radiolabeling of natural peptide ligands to cancer-associated receptors. Peptides are defined as short chains of maximally 70 amino acids but are most of the time smaller and generally have a molecular weight below 2000Da. Peptide receptor radionuclide therapy (PRRNT) has been established in neuroblastoma patients that suffer from residual or metastatic disease after surgery. Seventy per cent of neuroblastoma tumors express somatostatin receptors on the cell surface, which can be targeted by octreotide analogs. Patients suitable for PRRNT with ^90^Y/^177^Lu-octreotide are identified with ^111^In-octreotide scintigraphy, and more recently using Gallium-68 (^68^Ga)-octreotide for PET/CT [22]. These diagnostic techniques enable the prediction of efficacy and the assessment of response to the treatment. Clinical data demonstrate that responses are observed in almost 50% of the patients [23]. Adverse effects are generally observed in the kidneys, as these organs act as the major excretion route. Renal accumulation of small radiolabeled peptides and proteins is a well-described phenomenon. Charge-related interactions between peptides and proteins with the tubular cells of the kidney lead to extensive re-uptake after glomerular filtration [24]. Consequently, strategies to reduce the kidney uptake of radiolabeled protein- or peptide-based vectors have been investigated intensively. For instance, the interaction of peptides and proteins with the renal tubular cells can be reduced by the administration of an excess of cationic amino acids. Moreover, many manuscripts report reduced kidney retention using the plasma expander Gelofusin. This plasma expander consists of succinylated bovine gelatin molecules and has been shown to enhance excretion of megalin ligands. Kidney retention of radiolabeled octreotide analogs is reduced with ∼ 45% using an infusion of Gelofusin [25,26]. A combination of Gelofusin and l-lysine could reduce kidney uptake even further [27,28]. Recently, nephrotoxicity was analyzed using renal clearance data of 74 patients with gastroenteropancreatic neuroendocrine tumors, undergoing PRRNT with ^177^Lu-octreotate that was confused with cationic amino acids. Serious nephrotoxicity after PRRNT with ^177^Lu-octreotate was rare (1.3%). Slight renal impairment could be detected in 43% of cases using ^99m^Tc-DTPA clearance assessments. Consequently, researchers concluded that cumulative administered activity of ^177^Lu-octreotate is not a major determinant of renal impairment [29]. Although PRRNT has been proven very successful in the treatment of cancer, it is strictly dependent on the presence of tumor-associated antigens and the availability of targeting peptide ligands. Apart from somatostatin analogs, other peptides are under investigation for PRRNT, including vasoactive intestinal peptide (neuroendocrine tumors), cholecystokinin (medullary thyroid carcinomas, small cell lung cancers and stromal ovarian cancers) and bombesin analogs (carcinomas of breast, prostate and colon) [30-32].

## 5. Protein scaffolds of non-immunoglobulin origin in TRNT

Recent advances in TRNT have been complemented by the focus on alternative man-made protein formats, referred to as ‘scaffold molecules’. Over 50 different protein scaffolds have been reported such as Affibodies, DARPins, anticalins, knottins, adnectins and monobodies [33-36]. In general, large banks of a stable protein scaffold are generated by randomization of amino acids in solvent-exposed areas, followed by a stringent selection procedure to retrieve binders from such libraries with desirable characteristics.

A protein scaffold that has been evaluated intensively for molecular imaging but also for TRNT is the Affibody molecule. Affibodies (7 kDa) originate from the Z-domain, which is derived from staphylococcal surface protein A [37]. Several manuscripts on anti-HER2 Affibody molecules highlight their strong potential for the diagnosis of cancer [38-40]. In one study, a bivalent Affibody (Z_HER2:342_)_2_ has been fused to an albumin-binding domain (ABD), which interacts with serum albumin and therefore prolongs the blood residence time of the construct. Experimental TRNT of micro-xenografted mice with about 20 MBq of ^177^Lu-DTPA-ABD-(Z_HER2:342_)_2_ leads to a complete prevention of tumor formation [41]. No data are presented on ^177^Lu-labeled Affibody molecule without fusion to an ABD and thus without extended blood residence time. In addition, the biodistribution of anti-HER2 Affibody (Z_HER2:4_)_2_, labeled with Astatine-211 (^211^At) using the precursor N-succinimidyl-*p*-(trimethylstannyl)benzoate, was evaluated in HER2^+^ SKOV3 tumor xenografted mice upon co-administration with l-lysine and Na-thiocyanate. Here, researchers noted high doses that were delivered to thyroid (17.24 ± 51.38 Gy/MBq), lung (3.78 ± 0.71 Gy/MBq) and spleen (4.09 ± 1.03 Gy/MBq) and only 2.05 ± 0.19 to tumor. In terms of *in vivo* nonspecific accumulation and stability, the presented method for ^211^At-labeling might not be ideal for TRNT [42].

Jiang and coworkers [43] described the generation of cystine knot peptides (knottins) binding integrin receptors that are overexpressed on the surface of a variety of malignant human tumor cells and tumor neovasculature. Knottins 2.5 D and 2.5 F were radiolabeled with ^177^Lu, via a DOTA chelator, as novel scaffold molecules for TRNT. Biodistribution measurements showed specific tumor uptake, but again with high renal accumulation as the dose-limiting factor [43].

In conclusion, only few man-made scaffold proteins have been investigated in depth for TRNT. Some of them, such as Affibodies, show great potential but additional (pre-) clinical work is definitely required.

## 6. Nanobodies: a new theranostic tool in cancer treatment

### 6.1 Nanobodies

Nanobodies are derived from a unique antibody format that is present in the family of *Camelidae*. These species possess besides conventional antibodies, an additional antibody class that is devoid of light chains and that is referred to as heavy-chain-only antibodies (HCAbs) [44], as shown schematically in Figure 1. The heavy chain of these HCAbs lacks the constant domain C_H_1, which is responsible for pairing with C_L_. The single, variable, antigen-binding domain of the HCAbs, which is referred to as V_H_H, associates to its cognate target via three loops only. Functional, *in vivo* affinity-matured V_H_H fragments (13 – 14 kDa), also known as nanobodies or camel single-domain antibody fragments, are easily cloned from immunized camels or llamas and selected by phage display panning techniques. Consequently, nanobodies can be generated against a plethora of markers. Due to their small paratope, nanobodies target cryptic or hidden antigens that are inaccessible to larger antibodies [45].

The V_H_H fragment has undergone important adaptations in comparison to a V_H_ of a conventional antibody. A larger region around the first antigen-binding loop (CDR1) is hypervariable [46] and the third antigen-binding loop (CDR3) is on average longer than the corresponding loops from conventional V_H_s. In dromedary V_H_Hs, an extra disulfide bridge often stabilizes these enlarged loops [47]. The framework-2 region contains several polar residues [48] attributing to the generally higher hydrophilicity and solubility [49] of nanobodies compared to single domain V_H_ or V_L_ domains from conventional mAbs.

Moreover, nanobodies resist well against chemical or thermal denaturation and might refold reversibly to their native conformation [50]. They are cloned and produced efficiently in both *Escherichia coli* and yeast [51,52].

Nanobodies share a high degree of sequence identity with human V_H_s and are considered low immunogenic, as demonstrated in mice [53]. However, whenever it should be needed, a V_H_H can still be further ‘humanized’. Vincke and coworkers described a general strategy to produce a humanized nanobody scaffold that manages to accommodate antigen-binding loops of various nanobodies to graft the antigen specificity of the loop donor [54]. This work inspired Vaneycken and colleagues to humanize an anti-CEA nanobody without affecting its tumor targeting capacity [55].

Usually, an intrinsic affinity in the (sub)nanomolar range is observed for nanobodies and the antigen binding site of mAbs to their cognate antigen. However, in contrast to monovalent nanobodies, the affinity of mAbs having two antigen-binding sites per molecule might improve from avidity effects. Due to their single domain character, however, nanobodies are formatted easily into multivalent or multispecific constructs. Bivalent nanobodies (Figure 1) have been shown to exhibit higher avidity towards their target, leading to an improved retention at sites of antigen expression (Figure 1) [56]. Roovers and coworkers described increased absolute tumor uptake of bivalent and bispecific anti-EGFR nanobody constructs. Here, a biparatopic anti-EFGR nanobody showed an improved affinity for its target and an inhibition of tumor proliferation [57]. Moreover, the conjugation of the nanobody to an ABD (Figure 1) prolongs significantly the blood residence time and the fraction that accumulates in the tumor [58].

### 6.2 Nanobodies in nuclear imaging

The potential of nanobodies as *in vivo* diagnostic tracers in cancer is well documented. In addition, nanobodies against vascular cell adhesion molecule-1 [59] and oxidized LDL receptor-1 [60] have been evaluated for molecular imaging of artherosclerosis. Nanobodies were used also to image joint inflammation in rheumatoid arthritis [61,62] and specific immune cell types [63].

**Figure 2.**
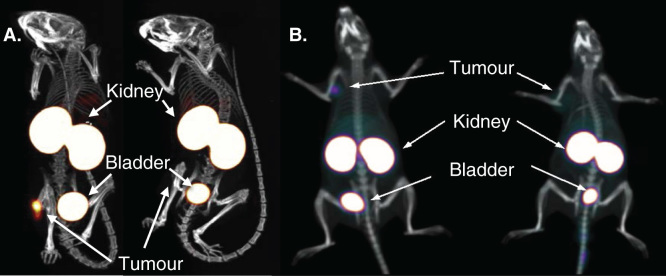
**Specific diagnostic tumor imaging with nanobodies at 1 h post-injection.** **A.** SPECT/CT images of mice injected with ^99m^Tc-labeled anti-HER2 nanobody. **B.** PET/CT images of rats injected with ^68^Ga-labeled anti-HER2 nanobody. Animals on left carry HER2-positive-xenografted tumors, animals on right carry HER2-negative-xenografted tumors.

In terms of nuclear imaging of cancer, nanobodies have been developed to target a variety of extracellular cancer cell biomarkers, such as CEA [55], EGFR [64], HER2 [65] and prostate-specific membrane antigen [66]. Absolute tumor uptake values of ^99m^Tc-labeled nanobodies were generally lower (ranging between 5 and 10 %IA/g at 1 – 3 h post-injection (p.i.) and gradually decreasing in time) than those obtained with mAbs at peak time points, but a 10-fold higher specific contrast was generated as early as 1 h post-injection. Radiolabeled nanobodies are cleared fast from the blood, yielding biphasic blood curves. Calculated blood half-lives of the initial phase are situated around 1 min and those of the slow phase around 30 min. At 1 h p.i., the percentage of injected activity per total blood volume generally decreases below 0.5. For clinical translation, the development of PET tracers is of interest, because of the superior image resolution and quantitative properties of clinical PET versus SPECT. Due to the short biological half-life of nanobodies, PET radioisotopes like ^68^Ga and Fluor-18 (^18^F) are favorable. In this regard, an anti-EGFR nanobody [67] and an anti-HER2 nanobody [68] were radiolabeled with ^68^Ga. Moreover, the first clinical trial with a ^68^Ga-labeled nanobody targeting HER2 in breast cancer patients is currently ongoing (EudraCT 2012-001135-31). SPECT/microCT images of xenografted mice injected with ^99m^Tc-labeled anti-HER2 nanobodies and PET/CT images of xenografted rats injected with ^68^Ga-labeled anti-HER2 nanobodies are shown in Figure 2.

**A.** Adapted from [21].
**B.** Obtained from [68]. PET: Positron emission tomography.

Movahedi *et al.* reported the generation of an anti-macrophage mannose receptor (MMR) nanobody for non-invasive imaging of tumor-associated macrophages [56]. The MMR is a transmembrane glycoprotein, expressed on macrophages [69]. It has been reported that certain components of the tumor stroma, especially in the hypoxic areas, highly over-express MMR and have dominant roles during tumor growth and processes like angiogenesis, metastasis and immune suppression.

Finally, a recent paper reported the development and characterization of nanobodies targeting the hepatocyte growth factor (HGF) in tandem with Zirconium-89 (^89^Zr) for PET imaging of HGF expression [70]. HGF and its receptor c-Met are associated with increased aggressiveness of tumors and poor prognostic outcomes for patients with cancer.

### 6.3 Improving pharmacokinetics of nanobodies for TRNT

In general, radiolabeled nanobodies are characterized by fast clearance through kidneys, resulting in moderate absolute tumor uptake and intense renal accumulation. Both could affect the effectiveness of TRNT. Increased absolute tumor uptake might be achieved by generating bi- or multivalent nanobody constructs, as previously described in Section 6.1. and schematically shown in Figure 1, or by increasing the hydrodynamic radius of molecular vectors to values above the renal treshold.

The effect of fusing an ABD [71] to ^177^Lu-labeled anti-EGFR nanobody [58] and ^89^Zr-labeled anti-HGF nanobodies [70] (Figure 1) on blood and kidney retention and tumor targeting have been explored. ABD fusion to bivalent anti-EGFR nanobody increased both blood residence time and tumor uptake levels and concomitantly reduced renal retention. Hence, tumor-to-kidney ratios rose from 0.03, 0.02 and 0.013 at 6, 24 and 72 h p.i. for the bivalent anti-EGFR nanobody to 1.7, 3.2 and 3.5 for the bivalent anti-EGFR nanobody fused with an ADB, in A431 tumor-xenografted mice [58]. However, the albumin-binding affinity also increased the radiation exposure to blood, BM and to additional highly perfused organs dramatically. Moreover, the extended blood residence time might raise the immunogenicity of nanobodies.

As mentioned in Section 4, renal retention of small proteins and peptides is predomantly dictated by charge-based interactions with the megalin/cubulin system in the renal tubuli. Gainkam and coworkers showed that co-administering a mixture of 150 mg/kg Gelofusin and 1.2 g/kg l-lysine reduced renal uptake of a ^99m^Tc-labeled anti-EGFR nanobody with 45%, as the tumor-to-kidney ratio went up from 0.03 to 0.07 at 1.5 h p.i., in A431 tumor-xenografted mice [72]. Another approach to reduce renal retention of nanobodies consists of mutagenizing the protein sequence at sites that are responsible for interaction with the megalin/cubulin system. In this regard, we described that polar residues in the nanobodies’ C-terminal amino acid sequence are predominantly responsible for renal retention. The administration of untagged ^177^Lu-labeled anti-HER2 nanobody co-infused with Gelofusin leads to a drop of 95% in renal retention as compared to nanobodies with a highly charged C-terminal amino acid tag, without affecting the tumor targeting capacity. Untagged ^177^Lu-labeled anti-HER2 nanobody noted tumor-to-kidney ratios of 0.63, 0.63, 0.83, 1.04, 1.5 and 2.88 at 1, 6, 24, 48, 72 and 168 h p.i.,respectively, in HER2^+^ SKOV3 tumor-xenografted mice [73]. This was also noted for an untagged ^68^Ga-labeled anti-HER2 nanobody, which revealed a 50% drop in renal accumulation at 1 h p.i. as compared to a hexahistidine-tagged equivalent. The tumor-to-kidney ratio noted 0.03 for the hexahistidine-tagged nanobody versus 0.1 for the untagged counterpart [68]. A similar observation was made by Wällberg and coworkers, as they demonstrated that amino acid substititions in the C-terminal amino acid sequence of an Affibody molecule had a significant impact on the extend of kidney retention [74].

### 6.4 Nanobodies in TRNT of the EGFR (ErbB family)

**Figure 3.**
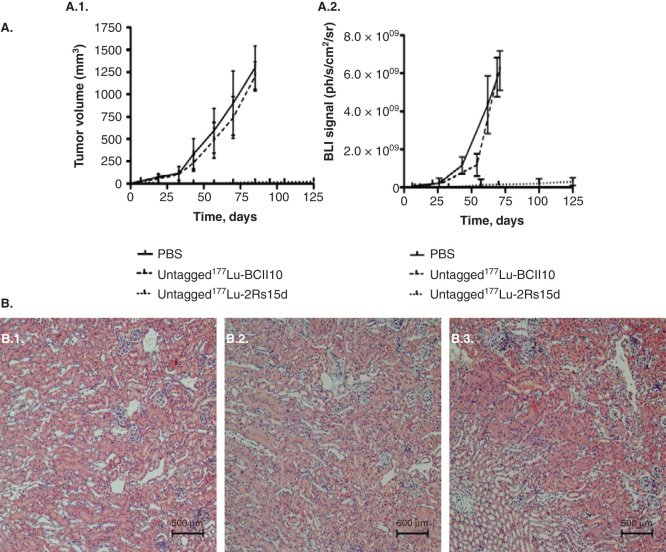
**TRNT using a ^177^Lu-labeled anti-HER2 nanobody in HER2^+^ SKOV3 tumor-xenografted mice (subcutaneous, right hind leg, 20 – 30 mm^3^ at start of TRNT); A. Tumor growth monitoring during TRNT.** Tumor volumes were quantified using **(A.1.)** caliper measurements (mm^3^) and **(A.2.)** bioluminescence imaging (ph/s/cm^2^/sr) as a function of time (days). Control groups (n = 8 per group) received either PBS or ^177^Lu-labeled non-targeting nanobody BCII10 (19.3 ± 0.8 MBq). Animals in the treatment group (n = 8) received a weekly i.v. injection of untagged ^177^Lu-labeled anti-HER2 nanobody 2Rs15d (20.7 ± 0.4 MBq). All treatments occurred with a 150 mg/kg Gelofusin co-injection. In terms of tumor growth, important differences were observed between the control groups and the treated group, for both caliper and bioluminescence measurements. **B.** Renal histopathology of ^177^Lu-dosed animal groups was compared to the PBS-treated animal group, 3 months after TRNT initiation. Sections were H&E stained and examined for signs of renal toxicity. No differences in renal histology were observed between the animal groups that received **(B.1.)** PBS, **(B.2.)** ^177^Lu-labeled BCII10 and **(B.3.)** ^177^Lu-labeled 2Rs15d.

In a first attempt to assess the potential of radiolabeled nanobodies for targeted cancer therapy, monovalent nanobodies targeting HER2 were labeled with ^177^Lu using DOTA-based macrocyclic and DTPA-based acyclic bifunctional chelators. A good absolute tumor uptake combined with low background uptake was observed using specific DTPA-based conjugates [75]. Thereafter, a first report was published on TRNT using an untagged ^177^Lu-DTPA-labeled anti-HER2 nanobody (10.1 ± 0.2 MBq), co-infused with Gelofusin, in HER2^+^ SKOV3 tumor-xenografted mice. Dosimetry calculations revealed a dose of 0.90 Gy/MBq that was delivered to both tumor and kidneys and extremely low doses (< 0.05 Gy/MBq) to healthy tissues [73]. In a comparative study, ^177^Lu-DTPA-Trastuzumab (anti-HER mAb) supplied six times more radiation to the tumor than untagged ^177^Lu-DTPA-nanobody, but concomitantly also a 155-, 34-, 80-, 26- and 4180-fold higher radioactivity burden to the lung, liver, spleen, bone and blood, respectively. Nanobody-based TRNT in mice-bearing small established HER2^pos^ tumors (tumor volume of 20 – 30 mm^3^) led to an almost complete blockade of tumor growth and a significant difference in event-free survival between the treated and the control groups. Based on histology analyses of kidney samples, no evidence of renal inflammation, apoptosis or necrosis was observed, as shown in Figure 3 [73]. However, kidneys are characterized as late response tissues in terms of signs of toxicity. Therefore, an adequate investigation of toxicity beyond 6 months will be needed. Moreover, kidney function can be monitored during TRNT using chrome-51 (^51^Cr) EDTA or ^99m^Tc-DTPA.

Figure obtained from [73]. i.v.: Intravenous; TRNT: Targeted radionuclide therapy.

In another study, bivalent constructs of anti-EGFR nanobodies fused to an ABD were generated and radiolabeled with ^177^Lu using a DOTA-derivative [58]. Extended blood clearance half-life and higher tumor uptake were clearly noticed by fusing the ABD to the nanobody. No dosimetry analysis was performed, which makes it difficult to estimate the radiation dose delivered to tumor and healthy tissues as a result of the extended blood half-life.

Pruszynski and coworkers reported the radio-iodination of a cysteine-tagged anti-HER2 nanobody (5F7GGC) with ^125/131^I, comparing iodination through the conventional IODO-GEN method with labeling via the residualizing agent Nϵ-(3-[131I]iodobenzoyl)-Lys5-Nα-maleimido-Gly1-GEEEK (IB-Mal-DGEEEK) [76]. This residualizing agent contains multiple negatively charged D-amino acids and would therefore enhance cellular retention of radioactivity after receptor-mediated internalization. Improved stability and tumor retention were observed. However, due to the residualizing character of the prosthetic group, very high uptake values in kidneys were noted. In a second manuscript, the same group compared the two methods of radio-iodination with a third approach involving the prosthetic group N-succinimidyl 4-guanidinomethyl-3-iodobenzoate (SGMIB) [77]. Radio-iodination of the anti-HER2 nanobody using SGMIB resulted in a reagent with considerably improved targeting properties *in vitro* and *in vivo* compared with nanobody, radiolabeled according to the IODO-GEN and IB-Mal-D-GEEEK methods, as highlighted by higher tumor retention in combination with much lower uptake in healthy tissues including kidneys. SGMIB was found superior, underscoring the importance of matching the labeling method with the normal-tissue clearance and tumor catabolism properties of the protein molecular carrier. Tumor-to-kidney ratios for ^125^I-SGMIB-nanobody (93 kBq) were < 0.5, < 0.5, 1, 2 and 3 at 1, 2, 4, 8 and 24 h p.i. in HER2^+^ BT474/M1 tumor-xenografted mice [77]. These recent successful progresses emphasize the theranostic potential of nanobodies in cancer, as they demonstrate their potential utility with ^124^I, ^123^I and ^131^I for PET and SPECT imaging and for TRNT, respectively.

Taken together, these novel findings highlight the potential of radiolabeled nanobodies in TRNT. The choice of nanobody format, as well as the radiolabel can have a major impact on both tumor uptake and kidney retention, the latter being generally considered the dose-limiting organ.

### 6.5 Nanobodies in multiple myeloma: the road to personalized medicine

**Figure 4.**
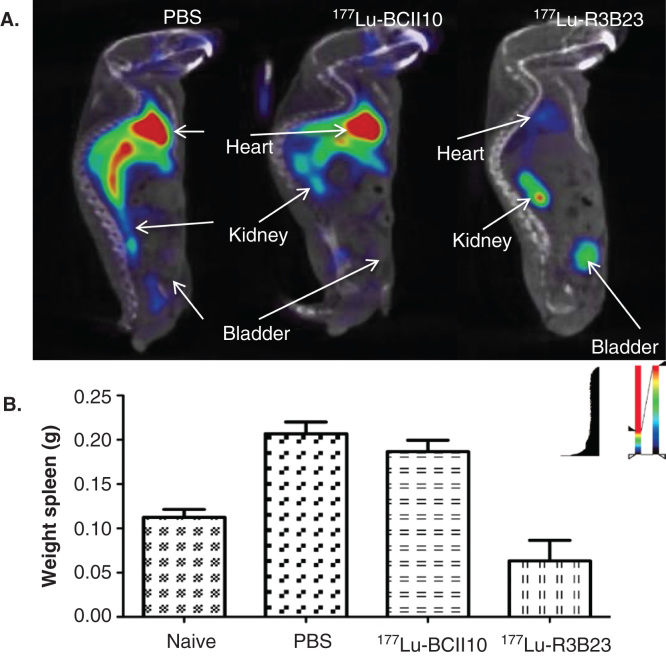
**Prophylactic TRNT using ^177^Lu-labeled nanobody R3B23, which targets 5T2 multiple myeloma (MM) cell-produced M-protein.** Syngeneic mice were i.v. injected with 2 × 10^6^ 5T2MM cells and TRNT started 1 week after tumor cell inoculation. These 5T2MM mice received a weekly i.v. injection of either PBS, 18.5 MBq ^177^Lu-labeled non-targeting nanobody BcII10 or 18.5 MBq ^177^Lu-labeled R3B23. **A.** Sagittal SPECT/micro-CT images 1 h after i.v. injection of ^99m^Tc-R3B23 Nanobody in mice receiving TRNT for 5 weeks. 5T2MM mice that received ^177^Lu-R3B23 TRNT showed lower levels of circulating M protein that was captured by the ^99m^Tc-R3B23 radiotracer than in control groups, a sign of delayed disease progression. **B.** Weights of spleens (homing site of 5T2MM tumor cells) after 7 weeks of TRNT with ^177^Lu-R3B23 or controls.

Adapted from [79]. i.v.: Intravenous; TRNT: Targeted radionuclide therapy.

Multiple myeloma (MM) is characterized by the monoclonal expansion of malignant plasma cells in BM and by production of monoclonal protein (M-protein). M-protein or paraprotein is a mAb that is secreted into the bloodstream thereupon associating with the MM cell surface in a significant fraction of patients [78]. Recently, nanobodies were generated against the paratope of M-protein (i.e., anti-idiotypic) produced by 5T2 MM cells, as a tool to image 5T2 MM cancer progression using SPECT/microCT [79]. The 5T2 MM model is a syngeneic, immunocompetent model that resembles human MM clinically and biologically. Radiolabeled with ^99m^Tc, these generated nanobodies were able to monitor disease progression non-invasively by targeting MM cells in minimal residual disease. Moreover, TRNT using a ^177^Lu-labeled anti-5T2 MM nanobody led to an inhibition of disease progression in treated mice compared to control animals, as illustrated in Figure 4. In this study, treated mice had significantly less circulating M protein. Moreover, the weight of the spleen (the homing site of 5T2 MM cells) in treated mice was similar to that in healthy mice, in contrast to control groups where the spleen was significantly enlarged (due to severe disease progression). Consequently, these findings provide evidence for the successful use of nanobodies in the development of novel diagnostic and therapeutic techniques in MM. Furthermore, it suggests that patient-tailored therapy might become feasible. As the generation of anti-idiotype nanobodies is a straightforward process, it should be possible to identify patient-tailored anti-idiotypic nanobodies during the period of first-line therapy. Personalized therapeutic radiolabeled nanobodies could then be applied when complete remission is achieved in order to slow down or stop resurgence of minimal residual disease.

## 7. Expert opinion: the future role of radiolabeled nanobodies in cancer therapy

The integration of diagnostic testing for the presence of a molecular target is of interest and allows the prediction of successful TRNT. The streamlined identification of nanobodies with excellent specificity against a variety of tumor-associated antigens is well established, allowing the development of radiolabeled vehicles for use in a generic personalized healthcare strategy in oncology. The recent preclinical progress, both in diagnosis and TRNT, confirms their value as theranostic tools in cancer treatment.

- Nanobodies, similar to certain peptides and artificial protein scaffolds, exhibit fast diffusion and clearance kinetics in organisms, specific accumulation in target tissue combined with low nonspecific accumulation. Hence, high-contrast SPECT or PET images are generated as fast as 1 – 3 h p.i., thereby providing an early patient diagnosis. Accordingly, diagnostic scans can be generated using short-lived radioisotopes, which seriously reduce the exposure of patients to radiation. Imaging using mAbs, radiolabeled with for example ^89^Zr, takes several days until the desired contrast is obtained due to the slow blood clearance kinetics. Moreover, the EPR effect often leads to false positive results. Some of the issues with mAb-probes can be overcome by the genetic engineering into smaller mAb fragments. However, compared to nanobodies mAb fragments are frequently characterized by lower stability, slower clearance, suboptimal tumor targeting and/or undesirable uptake in healthy tissues, which often limits their usefulness as generic tracers for accurate diagnostic imaging.
- Radiolabeled nanobodies could be used for early diagnosis/identification of a specific tumor-associated biomarker. Early detection of a specific biomarker attributes to the selection of the appropriate treatment strategy per patient. A whole body scan generates much more information on the presence of a certain biomarker, not only in the primary tumor but also in metastasized lesions, compared to blind biopsies, which is the current standard practice in oncology. Consequently, imaging with nanobodies could be developed to act as a valuable decision-making step in cancer therapy.
- Nanobodies that are labeled with therapeutic radionuclides reach the tumor with high specificity and only extremely low uptake in healthy tissues is recorded in preclinical dosimetry calculations. In RIT settings, mAbs target the tumor with dosimetry values that are about fivefold higher, but at the expense of delivering a much higher dose to blood and highly perfused organs. Therefore, toxicity to these organs limits the actual radioactive dose that is delivered to the patient by mAb carriers. Genetically engineered mAb fragments display faster clearance kinetics, but for reasons stated above, tend to show lower tumor-to-background ratios and a less optimal dosimetry profile than nanobodies.
- Due to the monovalent character of nanobodies, they possess a lower avidity compared to mAbs and bivalent mAb fragments such as diabodies and minibodies. Bivalent nanobodies could potentially compensate for this. However, it is clear that the bivalent character of nanobodies should not hamper their beneficial pharmacokinetics and targeting capacities. Due to a lack of studies comparing monovalent versus bivalent nanobodies, this claim is yet to be confirmed.
- Ideally, the diagnostic and therapeutic nanobody probe should consist of the same format. Predictive SPECT of PET scans could then be applied for preliminary dosimetry estimations. PET tracers are preferred as they generate improved resolution, which is especially important for the identification of small lesions. In this regard, nanobodies that are radio-iodinated with ^124^I could serve for PET imaging and dosimetry purposes, whereas ^131^I could be applied in TRNT, without substituting the actual theranostic agent. However, ^18^F- and ^68^Ga-labeled nanobodies have already shown excellent preclinical and early clinical results and these radioisotopes’ half-lives more closely match the biological half-life of nanobodies.
- Nanobodies recognizing an epitope, non-competitive to that of a targeted unlabeled therapeutic compound, are easily identified from ‘immune’ nanobody libraries. This opens the possibility to use radiolabeled nanobodies as adjuvant therapies in combination with conventional targeted agents. Due to the exceptionally specific targeting capacity of radiolabeled nanobodies, nanobody-based TRNT can act as an add-on therapy to improved outcome of patients that receive mAb immunotherapy. This claim has also been suggested by Sharma *et al*. [80]. Radiation that targets signaling pathways in tumor cells can be viewed as immunosupportive vaccines that would liberate multiple neo-antigens that, in combination with immune-checkpoint blockade, might result in a multiprolonged and effective immune attack [80]. Nanobody-TRNT and immunotherapy could therefore be viewed as a synergic therapeutic combination.
- Nanobody-TRNT would most likely find its application in the treatment of micrometastatic and minimal residual disease, where the highly specific deposition of radioactivity to tumor cells is of upmost importance. However, the improved tumor penetration of nanobodies compared to mAbs and most of its derived fragments might open up the road to treating solid tumors. Much more research is, however, necessary to underscore the use of nanobody-based TRNT of solid tumors.
- Kidney retention of radiolabeled nanobodies is a major limiting factor for TRNT. However, recent studies indicate that this characteristic is reduced through adjustments in the nanobody C-terminal sequence and through a co-infusion with Gelofusin and/or positively charged amino acids. Long-term follow-up of potential kidney toxicity is yet to be done as kidneys are generally considered to be late response tissues. In addition, kidney function could be monitored during TRNT using ^51^Cr-EDTA or ^99m^Tc-DTPA.
- Fusing nanobodies with an ABD increases the blood circulation times of the construct, their absolute uptake values in tumors and tumor-to-kidney ratios. It would also reduce the necessity to perform repeated dosing. However, this strategy comes at the expense of an elevated delivery of radioactivity to blood and highly perfused organs. Further preclinical and clinical assessments should proof the added value of such nanobody constructs relative to monovalent nanobodies.
- The choice of radioisotope, as well as the method for radiolabeling can have an important effect on the biodistribution, as shown already for both ^177^Lu and ^131^I. Regarding nanobody-based TRNT, residualizing radionuclides are of highest interest, as receptor-mediated internalization would trap radionuclides inside the target cell and therefore prolong the exposure. Alternatively, non-residualizing halogens like ^131^I can be retained in target cells using residualizing prosthetic groups.
- α-emitting radioisotopes are characterized by high LET values. Due to the highly specific targeting capacity of nanobodies, the combination of nanobody and an α-emitter might deliver a highly specific toxic load to residual or micrometastasized cancer cells, while minimizing impairment to healthy cells. Many α-emitters are characterized by a fast decay, which would be beneficial in terms of dosimetry and would match the short biological half-life of nanobodies. The first study using nanobodies radiolabeled with α-emitting radioisotopes yet needs to be performed.
- Nanobodies are highly homologous to human V_H_. No immunogenicity in humans has been documented so far, which creates the opportunity of repeated treatment cycles. This is in contrast to murine mAbs, which are frequently used in RIT instead of humanized mAbs because of improved blood clearance and lower radiation toxicity, where repeated dosing enhances the risk of inducing antibody responses.

In conclusion, the use of radiolabeled nanobodies as theranostics in cancer is a fast growing concept. Recent preclinical data have highlighted their potential, but their impact in the clinic is yet to be demonstrated. Moreover, additional preclinical work remains to be performed, focusing on the optimal nanobody construct in combination with the evaluation of a variety of radioisotopes and chelator types, and their influence on dosimetry and therapeutic efficacy. It is, however, clear that there is an unmet need to specifically identify and treat minimal residual and micrometastatic disease after the conventional treatment options like surgery, chemotherapy and external beam radiotherapy. Based on their superior *in vivo* stability, their high specificity of targeting and the very low accumulation in healthy tissues compared to most evaluated mAbs and mAb fragments, we claim nanobodies could generate a new theranostic frame for cancer therapy where it could offer improved personalized medicine opportunities.

Early diagnostic scans using nanobodies would therefore serve as support for dose estimation and impact the rationalization of treatments based on dose-effect relationships. An initial low activity scan could be performed for dosimetry calculations, followed by a high therapeutic dose, calculated from the dosimetry analysis. We believe nanobodies for both diagnostic and therapeutic applications should be applied in the same monovalent format.

The first clinical trial with a ^68^Ga-labeled anti-HER2 nanobody as a molecular imaging tracer targeting the breast cancer marker HER2 is being finalized (EudraCT 2012-001135-31) and will generate the first in-human data on tolerability, dosimetry and its capacity to identify HER2 overexpressing lesions.

Article highlights.

- The integration of diagnostic testing for the presence of a molecular target is of interest to predict successful targeted radionuclide therapy (TRNT).
- Nanobodies are small variable domains of HCAbs from *camelidae* and are being evaluated intensively as diagnostic markers in both mouse models and clinical settings, especially in cancer.
- Recent preclinical advances in TRNT using nanobodies point out their potential for therapeutic applications.
- Nanobodies against all possible cellular targets are easily identified, emphasizing their potential as generic theranostic tools in cancer.

This box summarizes key points contained in the article.

## Declaration of interest

M D’Huyvetter is funded by the foundation Emmanuel van der Schueren. T Lahoutte is a Senior Clinical Investigator of the Research Foundation Flanders (FWO). The research at ICMI is funded by the Belgian State, Nationaal Kankerplan, Vlaamse liga tegen kanker, Stichting tegen kanker, FWO-Vlaanderen, IWT and Vrije Universiteit Brussel. The authors have no other relevant affiliations or financial involvement with any organization or entity with a financial interest in or financial conflict with the subject matter or materials discussed in the manuscript apart from those disclosed.
